# Gut Microbiota Composition and Sleep in Preschoolers: The ELFE Birth Cohort Study

**DOI:** 10.3390/children12091240

**Published:** 2025-09-16

**Authors:** Zeinab Houshialsadat, Cécile Zaros, Marie-José Butel, Marie-Aline Charles, Gaël Toubon, Sabine Plancoulaine

**Affiliations:** 1 Université Paris Cité, Institut National de la Santé et de la Recherche Médicale (INSERM), Institut National de Recherche pour l’Agriculture, l’Alimentation et l’Environnement (INRAE), Centre de Recherche en Epidémiologie and StatistiqueS (CRESS), F-75004 Paris, France; zeinab.houshialsadat@mail.utoronto.ca (Z.H.); marie-aline.charles@inserm.fr (M.-A.C.); gael.toubon@ki.se (G.T.); 2 Ecole des Hautes Études en Santé Publique (EHESP), F-35043 Paris, France; 3 Ined, Inserm, EFS Unité Jointe Elfe, F-93322 Aubervilliers, France; cecile.zaros@inserm.fr; 4 Université Paris Cité, Institut National de la Santé et de la Recherche Médicale (INSERM), UMR-S 1139, Fonction Placentaire et Reproductive, Microbiote Pré & Postnatal (FPRM), F-75006 Paris, France; marie-jose.butel@u-paris.fr; 5 Université Claude Bernard Lyon 1, Centre National de la Recherche Scientifique (CNRS), Institut National de la Santé et de la Recherche Médicale (INSERM), Centre de Recherche en Neurosciences de Lyon (CRNL) U1028 UMR5292, F-69500 Bron, France

**Keywords:** gut microbiota, diversity, composition, sleep duration, night waking, sleep onset difficulty, preschool

## Abstract

Background/Objectives: Sleep is essential for children’s well-being, yet insufficient sleep duration and quality are common among preschoolers. The brain–gut microbiota axis, a bidirectional communication network connecting the brain, the gastrointestinal tract, and the microorganisms living there, known as the gut microbiota, influences sleep regulation, but its role in children remains largely unexplored. Here, we examined the association between gut microbiota and sleep in preschoolers from Étude Longitudinale Française depuis l’Enfance (ELFE) birth cohort study. Methods: This study included 597 children (51.2% boys) with available stool samples and sleep data at 3.5 years. The gut microbiota data was analyzed using bacterial 16S rRNA sequencing. Data on day and night sleep durations and frequencies of sleep onset difficulties and night waking were collected through telephone questionnaires and grouped into ‘optimal’ and ‘suboptimal’ clusters using Latent Class Analysis. Statistical analyses involved multivariate logistic regressions or multivariate permutation analysis of variance, controlling for confounders. Results: In total, 25% of the included children were in the suboptimal sleep cluster. No significant associations were found between gut microbiota diversity and composition and sleep clusters at age 3.5 years. Similarly, no differences were found in the abundance of specific microbiota genera between the two sleep clusters. Conclusions: While emerging evidence suggests correlations between gut microbiota and sleep in preschool children, our results do not confirm such correlations. The data used in this study were obtained from a homogeneous, high socioeconomic population, which must be considered when interpreting the findings. Further research is needed to validate the results of this study.

## 1. Introduction

Sleep is a natural physiological condition of unconsciousness that is essential to children’s health and well-being [1]. Sleep characteristics mainly evolve in the early years of life, highlighting the significance of sufficient sleep duration and quality for children’s physical and mental health development and maintenance [2,3]. Children of preschool age (3–5 years) are prone to sleep complications, including short sleep duration, sleep onset difficulties and night waking [3,4]. Emerging evidence suggests that insufficient quantity and/or quality of sleep can negatively impact children’s health, including well-being, growth, cognitive function, and academic success [5,6,7,8].

While sleep duration is age-specific, the overall decline in sleep duration in early years of life has been contributing to sleep deprivation among contemporary children [9]. Analysis of data from the Zurich Longitudinal Study involving 493 children and adolescents showed a 40 min reduction in total sleep duration per 24 h, mainly due to delayed bedtime [10]. Also, in a cross-sectional study involving 4321 Italian children and adolescents, 71.5% of preschoolers did not meet the age-specific recommendations for total sleep duration [11]. In the same study, sleep onset difficulties and frequent night awakenings per week were the most prevalent sleep disorders [11]. In a cohort study involving 11,500 children in England, night waking was present in 23% of children at 6 months, 50% at 18 months, and 49% at 3 years [9]. Additionally, in the Etude des Déterminants pré et post natals du développement et de la santé de l’ENfant (EDEN) study involving 1346 children in France, the prevalence of night waking (>2 nights per week) increased by 4% from 2 to 3 years [12]. In a study of 2889 children in Italy, night waking was present in about 35% of toddlers aged 6 to 14 months [13]. In the same study, sleep onset difficulties were found in 11% of toddlers aged 6 to 12 months, 7.5% of toddlers aged 13 to 24 months, and 4.7% of toddlers aged 25 to 48 months [13]. Although insufficient sleep duration can independently occur in preschool years, it is often exacerbated by both sleep onset difficulty and night waking [12]. Sleep disturbances and insufficient sleep duration that begin from early years often persist during childhood, adolescence and up to adulthood if not adequately addressed [12,14,15,16]. Thus, studying the early determinants of sleep is warranted to better understand sleep physiopathology and develop strategies to improve sleep in childhood and adulthood.

Link between the gut and the brain has been known since the 17th century [17]. A gut–brain bidirectional link and a role for the gut microbiota in sleep physiology have been suggested [18,19]. Indeed, the microbiota contributes to signaling by providing metabolites and bioactive molecules including neurotransmitters [20]. Conversely, these neurotransmitters can influence the gut microbiota community. In addition, bacterial metabolites as short-chain fatty acids may alter the gut barrier function which can contribute to neuroinflammation and neurodevelopement disorders [21]. The perinatal period is an important step and emerging evidence indicates that maternal prenatal gut microbiota as well as early bacterial colonization can influence offspring neurodevelopment, modulating neuroimmune maturation and brain development through the gut–brain axis [21]. The initial establishment of the gut microbiota in infants occurs at birth [22,23,24] and its development continues until reaching an adult-like composition around the age of 3 years, though the age window is debated upward (approximately up to 6 years) [25,26,27].

Although the Developmental Origins of Health and Disease (DOHaD) theory suggests that the first three years of life set the stage for the development of both healthy gut microbiota and sleep patterns [28,29], a small handful of studies have focused on their potential link in preschoolers (aged 3–5 years), and the findings are inconsistent. Thus far, only one study in Canada has investigated the gut microbiota–sleep link in preschoolers [30]; however, this study did not consider the potential role of confounders. Given the scarcity of evidence in this field and the novelty of this research area during the preschool age, this study aims to assess the association between the gut microbiota (diversity and composition) and sleep (night and day sleep duration, sleep onset difficulty, and night waking) in children aged 3.5 years from the French Longitudinal Study of Children (Étude Longitudinale Française depuis l’Enfance; ELFE) prospective birth cohort study.

## 2. Materials and Methods

### 2.1. ELFE Birth Cohort Study

The study population was derived from the ELFE perspective birth cohort study [31,32]. Newborns included in the ELFE study (*n* = 18,030) were born after 33 weeks of gestation in 349 randomly selected maternity units across mainland France during 25 selected days grouped in four periods over the year out of French holidays (early April, end June/early July, end September/early October, end November/early December). Mothers aged 18 years and older, who were literate in French, Arabic, Turkish, or English, and who did not plan to relocate outside France within three years of inclusion, were eligible to participate. A detailed protocol of the ELFE prospective cohort study is available elsewhere [31]. The study protocol and the collection of stools were approved by the Committee for the Protection of People Participating in Biomedical Research, the national advisory committee on information processing in health research, and French National Data Protection Authority.

### 2.2. Maternal and Child Data Collection

Health and medical data for mothers and newborns during pregnancy and postpartum were collected through self-reported questionnaires at birth or extracted from the obstetrical and pediatric medical records. Follow-up data were collected via telephone interviews with mothers or fathers at 2 months, and 1, 2, and 3.5 years postpartum. However, it was mostly mothers who were interviewed (>99%). At 3.5 years, stool sampling kits were offered during home visits to a subsample of ~3000 children who had already participated in a biological sampling phase at birth. If they accepted a sampling kit containing RNAlater® preservative (Enterome SA, Paris, France), families were provided with further instruction. The sample was kept at 4 °C before shipment by mail within 24 h. In total, 657 stool samples were returned.

### 2.3. Sleep Characteristics

Data on usual bedtime, wake-up time, and day sleep napping habits on weekdays and weekends were collected through telephone questionnaires from mothers at age 3.5 years. Day sleep duration was assessed by the following question at 3.5 years: “Does your child take a nap?” and “What is your child’s total nap duration?” for weekdays, weekend days, or holidays. Night sleep duration was assessed by the following question at 3.5 years: “Usually, at what time does your child go to bed?”. Mean day and night sleep durations per day were calculated over the week in hours and minutes. The frequency of night waking and sleep onset difficulties were also assessed by the following questions at 3.5 years: “How many nights did your child wake up during the night this week? (all nights, 3 to 6 nights per week, 1 to 2 nights per week, and never)” and “When you put your child in bed, does he/she have difficulties falling asleep? For example, he/she calls or cries for a long time over 30 min? (almost always, often, sometimes, and never).”

### 2.4. Gut Microbiota Analysis

Stool sample collection, DNA extraction and sequencing, and data management and analysis were conducted as previously described [33]. In summary, DNA extraction followed the International Human Microbiome Standards operating procedure [34], and DNA sequencing was performed using Illumina MiSeq technology (V3, 2 × 250 bp), targeting the V3-V4 primers (V3fwd: TACGGRAGGCAGCAG and V4rev: TACCAGGGTATCTAAT) regions of the 16S bacterial rRNA gene. Samples were rarefied to 10,637 reads for all downstream analyses to adjust for the uneven sampling depth, unless stated otherwise. Gut microbiota data were analyzed at the genus taxonomic level to avoid uncertainties at the OTU level.

Alpha diversity was estimated using the Chao1 estimate, which provides an estimate of species richness and Shannon index, which reflects overall community diversity by incorporating both richness and evenness in the distribution of taxa. Beta diversity was assessed by computing dissimilarity matrices using Bray–Curtis and Weighted UniFrac distances. Inter-individual variability of the microbiota was visualized based on dissimilarity matrices through Principal Coordinate Analysis (PCoA). Enterotypes, defined as gut microbiota communities based on similar gut microbiota composition, were generated according to Partitioning Around Medoids (PAM) enterotyping guidelines [35].

### 2.5. Covariates

The following covariates/confounders were selected based on early-life exposures previously associated with the gut microbiota at 3 years in the ELFE cohort [33] and a thorough literature review, and were mapped with a Directed Acyclic Graph (DAG; dagitty.net software v3.1) [36]: (1) maternal characteristics including birthplace in France (yes/no), education (≤secondary level, ≤Baccalaureat +2, and >Baccalaureat +2), pre-pregnancy body mass index (BMI), exposure to psychotropic medications during pregnancy (yes/no), age at birth, gestational age, child vaginal delivery (yes/no), and breastfeeding duration, (2) child characteristics including biological sex, number of siblings, exact age at stool sample collection, tobacco exposure from pregnancy until 3 years (yes/no), antibiotics intake between 2 and 3 years (never, once, >once), main mode of care at 2 years (family, child minder, collective care), BMI Z-score measurement, and diet at 2 years (healthy and unhealthy dietary patterns, Table S1), and (3) household characteristics including income per consumption unit and pet ownership (yes/no) at 2 months, and residential setting at 3 years (urban/rural). All covariates were extracted from maternal interviews, except pet ownership that combined maternal and paternal information to limit missing data.

### 2.6. Statistical Analysis

All statistical analyses were performed in R (V 4.2.1). Children with similar sleep patterns in terms of sleep duration and sleep onset difficulty and night waking frequencies were clustered together using unsupervised Latent Class Analysis (LCA) [37]. For LCA purposes, sleep onset difficulties and night waking data were dichotomized (sleep onset difficulties: yes = almost always and often, no = sometimes and never; night waking: yes = ≥3 to 6 nights per week, no = <3 nights per week). Also, day and night sleep durations were recorded based on median cut-offs (day sleep duration ≤1 h 30 and >1 h 30 and nocturnal sleep duration ≤10 h 49 and >10 h 49). Two sleep clusters, ‘optimal’ and ‘suboptimal’, were identified based the minimum Bayesian Information Criterion (BIC) and clinical interpretability. The child was assigned to the cluster for which he/she had the highest probability of belonging to. Sleep clusters were used as outcomes in the present study.

Multivariable logistic regression models were used to assess the association between sleep clusters and alpha diversity metrics and enterotypes. Permutational Multivariate Analysis of Variance (PERMANOVA), based on 999 permutations, was used to test the association between the beta diversity metrices and sleep clusters. All statistical models analyzing the associations between the gut microbiota and sleep clusters were adjusted for the confounders presented in the covariates section.

Analysis of Differential Abundance Taking Sample Variation into Account (ALDEx2) [38] and Analysis of Composition of Microbiomes with Bias Correction (ANCOM-BC) [39] methods were used to identify specific genera associated with sleep clusters. Differential abundance testing methods were performed on non-rarefied data as both ALDex2 and ANCOM-BC have their own normalization methods. Benjamini–Hochberg’s False Discovery Rate (FDR) correction was applied to PERMANOVA, ALDex2, and ANCOM-BC analyses to account for multiple testing.

Missing values for covariates (2.4% of the sample) were imputed using the missForest random forest-based algorithm [40]. A sensitivity analysis was performed on complete-case data to assess the impact of missing data imputation.

## 3. Results

### 3.1. Study Population Description

**Figure 1:**
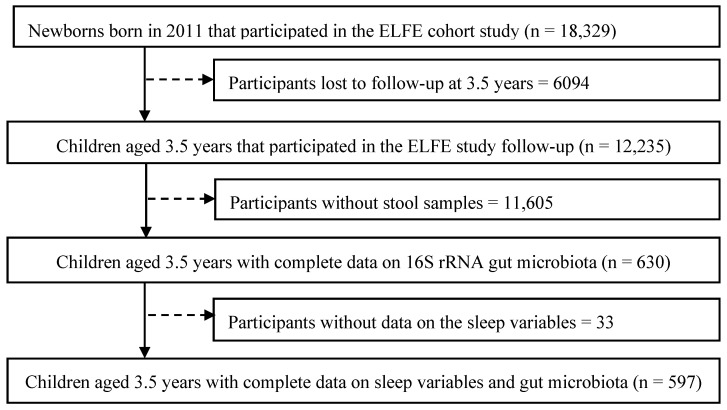
Study flow chart.

In total, 597 children aged 3.5 years (interquartile range [IQR], 3.4–3.6 years) with available gut microbiota and sleep data were included in this study (Figure 1). Compared to the excluded children, the included ones were more frequently boys (56.8% vs. 51.2%, *p* = 0.01) born in France (93.1% vs. 86.5%, *p* < 0.001). Included children had significantly older (32 vs. 31 years, *p* < 0.001) and more educated (50.3% vs. 36.2% with level > Baccalaureate +2 years, *p* < 0.001) mothers than those excluded. The average family income per consumption unit (CU) of the included cohort was significantly higher than that of the excluded group (44.6% vs. 32.8% with income >1944 euros/months/CU, *p* < 0.001). No other significant differences were observed among tested covariates.

### 3.2. Sleep Clusters Characterization

Two sleep clusters were identified as follows: ‘optimal’ and ‘suboptimal’. Table 1 shows the sleep characteristics of each cluster. The suboptimal cluster, comprising 25% of the children, was characterized by shorter night sleep duration and lower sleep quality compared to the optimal sleep cluster. Also, the suboptimal sleep cluster included more boys, children living in urban settings, children in collective care settings, and children without pets, compared to the optimal sleep cluster.

### 3.3. Gut Microbiota Diversity, Enterotypes, and Sleep Clusters

Gut microbiota richness and evenness were measured using the Chao1 estimate and Shannon index, respectively. As shown in Table 2, no significant association was found between the alpha diversity metrics and sleep clusters in the study population in both crude and adjusted models.

The gut microbiota profile of the study population was characterized by two distinct enterotypes dominated by Bacteroides (*n* = 488, 81.7%) or Prevotella (*n* = 109, 18.3%) genus and described previously in the ELFE cohort [33] and other birth cohorts [41,42]. As shown in Table 2, no significant association was found between the identified enterotypes and the sleep clusters.

### 3.4. Overall Gut Microbiota Composition and Differential Abundances Testing

**Figure 2:**
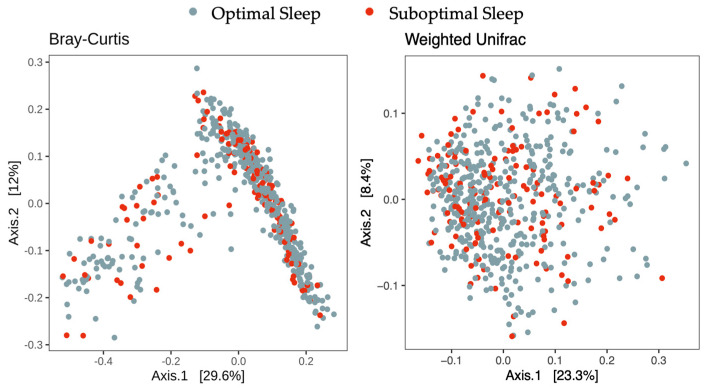
Gut microbiota community composition across the sleep clusters. Plots are based on the Bray–Curtis and Weighted Unifrac distances computed at the genus level.

Bray–Curtis and Weighted UniFrac distance measures were used to compute overall gut microbiota composition. Less than 1% of the total variance in the overall gut microbiota composition was explained by sleep clusters according to both distance measures. No significant association was found between the overall gut microbiota composition and sleep clusters (Table 3 and Figure 2).

Results from the ANCOM-BC differential abundance analysis showed that the gut microbiota of children in the optimal sleep cluster was more abundant in *Erysipelatoclostridium* and an unclassified genus from the *Ruminococcaceae* family (Table S2). However, results from the ALDEx2 analysis did not find significant differences in the abundance of any bacterial genus between the two sleep clusters. Since no significant overlap was observed between the two methods, no conclusions were drawn regarding the association between specific microbiota genera and the constructed sleep clusters.

### 3.5. Sensitivity Analysis

Overall, missing data did not alter the results of the present study, as the findings remained consistent in the imputed and complete-case models (Tables S3 and S4).

## 4. Discussion

In this study including preschoolers enrolled in the ELFE birth cohort study, no significant association was found between gut microbiota diversity and composition and sleep clusters, characterized by sleep duration and quality.

Our findings regarding alpha diversity are aligned with the only published study among preschoolers, including 143 Canadian children aged 4.37 years (±0.48, standard deviation [SD]) in the Alberta Pregnancy Outcomes and Nutrition (APrON) [30]. In this study, no differences in observed OTUs and Shannon diversity index were observed between children classified in the low and high groups of total night-time sleep, sleep efficiency, and wake-time after sleep onset [30]. Outside the preschool age window, our results align with findings from a cross-sectional analysis of the CHILD cohort study involving 619 Canadian infants, which reported no significant associations between alpha diversity indices and total sleep duration [43]. In contrast, a longitudinal study of 162 infants in Switzerland identified a negative relationship between the Chao1 estimate and Shannon index and sleep duration from age 3 to 12 months [44]. Findings from studies involving preschool or infant populations must be interpreted in light of the developmental origins of sleep and gut microbiota characteristics across these years. Sleep patterns during infancy are notably distinct, often marked by significantly longer sleep durations up to 12 months, which may limit the generalizability of findings to older age groups, including preschoolers.

The sleep–gut microbiota association is more extensively studied in adult populations, though the reported findings remain relatively inconsistent. Aligned with results of the present study, a randomized clinical trial including nine healthy young men (23.3 years ± 0.6, standard error [SE]) reported no significant differences in the OTU richness and Shannon evenness indices after two nights of partial sleep deprivation (4 h reduction of sleep opportunity/night) [45]. In another randomized clinical trial among 19 healthy American men aged 17–45 years, a lower amplicon sequencing variant richness was observed after three nights of severely restricted sleep (2 h of sleep/night) [46]. In contrast, a study in 22 healthy American men (22.2 ± 3.1, SD years) reported a positive association between alpha diversity metrics and sleep efficiency and only the Shannon diversity index was negatively correlated with sleep waking, measured by a 30-day actigraphy recording [18]. This finding was further supported by another study involving 28 healthy American subjects (17 men/11 women aged 29.8 years (±10.4, SE)) [47].

In the present study, the overall gut microbiota community composition was assessed by beta diversity and microbiota genus abundance. In the sample of preschoolers included in this study, no differences were observed in the overall gut microbiota community composition between the optimal and suboptimal sleep clusters in both unadjusted and adjusted models. Our findings were consistent with a clinical trial involving controlled sleep modifications in healthy American adults [46], as well as a trial focusing on sleep extension in a group of sleep-deprived adults aged 32.4 years (±4.6, SD) [48]. However, in the only study focusing on gut microbiota and sleep involving preschoolers in Canada [30], those with night sleep above median had a higher relative abundance of Bifidobacterium, Parabacteroides, and Turicibacter, compared to children with night sleep duration below median [30]. These results may suggest that gut microbiota diversity and composition modifications are different in acute induced sleep deprivation compared to habitual sleep patterns in free-living participants. Beyond the study design, discrepancies can also be explained by differences in sample size, demographics including country of origin and age, data collection and management methods, and statistical analysis models and confounders. Aligned with the present study, sleep data in the CHILD birth cohort study in 3-month-old infants used parent-reported questionnaires and included questions about day and night sleep habits per 24 h period [43], whereas in studies conducted in Canada and Switzerland, sleep data were collected by actigraphy for 24/24 h during 2 and 11 continuous days, respectively [30,44]. Although objective methods of sleep data collection may enhance the data precision, subjective tools remain dominant in large-scale epidemiological studies, thus maintaining the validity and quality of comparisons across the literature.

To the best of our knowledge, this study is the first one to leverage a national birth cohort data to assess gut microbiota diversity and composition among preschoolers in association with sleep clusters, while controlling for the effect of potential confounders. Our study has several strengths, including a relatively large sample size, providing enough statistical power to assess the research hypothesis and the impact of potential confounders. Also, the availability of a large set of covariates within the ELFE birth cohort study enabled us to adjust our statistical models for potential confounders and assess the role of effect modifiers. Although accounting for potential confounders in the multivariate models did not help with clarifying the association between gut microbiota and sleep, identifying these factors and assessing their potential effect can inform future research in this realm. Finally, in this study, sleep duration and quality were assessed simultaneously, providing a versatile and pragmatic measure of sleep as a whole, whereas most studies among children and adults focused on each attribute, distinctively. Although we did not find significant associations using LCA-constructed sleep clusters, future research is warranted to explore other statistical approaches to continue assessing sleep in a multifaceted manner.

This study also has limitations. Firstly, the sleep data were collected from parents through telephone interviews [32]. Night waking and sleep onset difficulties were those noticed and reported by parents and night sleep duration was calculated based on the bedtime and wake-up time. Together, this may have led to an overestimation of sleep duration and an underestimation of night waking and sleep onset difficulties. Although objective methods of sleep data collection can provide more accurate estimations, subjective tools remain a classical approach in data collection in large epidemiological studies due to cost and logistic challenges [2,9,49]. The consistency in the use of subjective sleep assessment tools in our study and the literature facilitates valid comparison of overall results. Sleep-related breathing disorders were not accounted for as data was not available. However, if they were present in our selected subsample (N = 560 children out of 12,235 participants aged 3.5 years), children with habitual sleep-related breathing disorders, and therefore disturbed sleep, would have been clustered in the suboptimal sleep group i.e., with the poorest sleep characteristics, not modifying our main results. Also, considering the gut microbiota data, our study is limited by the lack of repeated assessments of children’s gut microbiota, which hinders adjusting for the chronological microbiota changes and exploring the patterns of change within early childhood. Thus, further longitudinal studies on this topic are required to validate our findings. Using 16S rRNA sequencing limited the description of bacterial composition at the genus level but allowed comparison with the literature. A shotgun metagenomic sequencing approach would have provided deeper information on the functional metabolic capabilities and importance of the microbiome at the species level. Finally, our study population was mainly composed of households with high socioeconomic status, which may limit the external validity of our findings for households with lower socioeconomic attainments. In addition, the selected families may have a high health literacy and healthy practices and lifestyle. This may be reflected in the sleep duration of children in both clusters, which is in line with the American Academy of Sleep Medicine (AASM)’s recommendations (i.e., 10–13 h of sleep per 24 h during the preschool period). Therefore, we cannot confirm an association between gut microbiota characteristics and extreme sleep disturbances in our population at this point.

Our results did not support the hypothesized association between gut microbiota diversity and composition and sleep clusters in a sample of preschool children from the ELFE cohort study. This may be explained by the homogeneity of the study population in terms of socioeconomic status. Also, the sleep duration of children in both clusters was within the AASM’s recommended ranges, which may have hindered detecting major gut microbiota differences across the two clusters. Future research may consider studying sleep, characterized by sleep duration and quality, in more heterogeneous populations to validate our findings and advance this research area.

## Figures and Tables

**Table 1:** Overall characteristics of the study population across the sleep clusters (*n* = 597).

	Suboptimal Sleep (*n* = 150)	Optimal Sleep (*n* = 447)	Overall *(n* = 597)	*p*-Value
	Sleep Characteristics			
	*n* (%)	*n* (%)	*n* (%)	
Day sleep duration ^§^				0.85
≤1 h 30	82 (54.7)	240 (53.7)	322 (53.9)	
>1 h 30	68 (45.3)	207 (46.3)	275 (46.1)	
Night sleep duration ^§^				<0.001
≤10 h 49	119 (79.3)	185 (41.4)	304 (50.9)	
>10 h 49	31 (20.7)	262 (58.6)	293 (49.1)	
Sleep onset difficulty				<0.001
No	52 (34.7)	399 (89.3)	451 (75.5)	
Yes	98 (65.3)	48 (10.7)	146 (24.5)	
Night waking				<0.001
No	62 (41.3%)	447 (100%)	509 (85.3%)	
Yes	88 (58.7%)	0 (0%)	88 (14.7%)	
	Maternal Characteristics			
	*n* (%)	*n* (%)	*n* (%)	
Born in France	139 (92.7)	417 (93.3)	556 (93.1)	0.85
Education at 2 months				0.90
≤Secondary level	33 (22.0)	106 (23.7)	139 (23.3)	
≤Baccalaureat +2	39 (26.0)	118 (26.4)	157 (26.3)	
>Baccalaureat +2	78 (52.0)	223 (49.9)	301 (50.4)	
Vaginal child delivery	127 (84.7)	362 (81.0)	489 (81.9)	0.31
Exposure to psychotropic medications during pregnancy	6 (4.0)	10 (2.2)	16 (2.7)	0.25
	Mean (SD)	Mean (SD)	Mean (SD)	
Maternal age (years)	31.7 (4.18)	31.8 (4.27)	31.8 (4.25)	0.78
Pre-pregnancy BMI (Kg/m^2^)	23.2 (4.58)	23.4 (4.71)	23.3 (4.65)	0.76
Breastfeeding duration (months)	4.81 (6.20)	3.87 (5.04)	4.10 (5.33)	0.06
	Household Characteristics			
	*n* (%)	*n* (%)	*n* (%)	
Income at 2 months (€/month/CPU)				0.84
≤1500	51 (34.0)	159 (35.6)	210 (35.2)	
1500–1944	49 (32.7)	151 (33.8)	200 (33.5)	
>1944	50 (33.3)	137 (30.6)	187 (31.3)	
Pet ownership at 2 months	64 (42.7)	242 (54.1)	306 (51.3)	0.02
Residential setting at 3.5 years				0.04
Rural	43 (28.7)	130 (29.1)	173 (29.0)	
Suburban	42 (28.0)	169 (37.8)	211 (35.3)	
Urban	65 (43.3)	148 (33.1)	213 (35.7)	
	Child Characteristics			
	Mean (SD)	Mean (SD)	Mean (SD)	
Gestational age (weeks)	39.5 (1.57)	39.6 (1.27)	0.0538 (0.918)	0.31
BMI Z-score at 2 years	0.10 (1.18)	0.04 (1.01)	0.05 (0.91)	0.56
Diet at 2 years				
Unhealthy dietary pattern	0.04 (1.62)	−0.01 (1.55)	0.00 (1.56)	0.70
Healthy dietary pattern	−0.13 (1.52)	0.04 (1.39)	0.00 (1.42)	0.23
Age at 3 years (months)	42.3 (1.78)	42.3 (1.71)	42.3 (1.73)	0.96
	*n* (%)	*n* (%)	*n* (%)	
Sex; girl	50 (33.3)	208 (46.5)	258 (43.2)	<0.01
Children with sibling	75 (50.0)	250 (55.9)	325 (54.4)	0.22
Main mode of care at 2 years				0.07
Family	38 (25.3)	120 (26.8)	158 (26.5)	
Child sitter	76 (50.7)	254 (56.8)	330 (55.3)	
Collective care	36 (24.0)	73 (16.3)	109 (18.3)	
Tobacco exposure from pregnancy up to 3.5 years	53 (35.3)	159 (35.6)	212 (35.5)	1.00
Antibiotics intake between 2 and 3.5 years				0.65
Never	47 (31.3)	152 (34.0)	199 (33.3)	
Once	36 (24.0)	92 (20.6)	128 (21.4)	
More than once	67 (44.7)	203 (45.4)	270 (45.2)	

^§^ Categories based on the median cut-offs; CPU = Consumption Unit, SD = Standard Deviation; categorical variables expressed as frequency and proportion (*n* (%)); Chi-squared or student test depending on the type of variable.

**Table 2:** Gut microbiota alpha diversity measures, enterotypes, and sleep clusters (*n* = 597). The optimal sleep cluster is the reference.

	Crude			Adjusted ^§^		
	OR	95% CI	*p*-Value	OR	95% CI	*p*-Value
Alpha diversity metrics						
Chao1 estimate ^†^	0.98	0.81, 1.18	0.80	0.95	0.78, 1.17	0.64
Shannon index ^†^	1.01	0.84, 1.22	0.90	0.98	0.80, 1.20	0.84
Microbiota Enterotypes						
B_type ^‡^	—	—	0.88	—	—	0.96
P_type	1.04	0.64–1.67		0.99	0.59, 1.62	

OR = Odds Ratio, CI = Confidence Interval, B_type = *Bacteroides* enterotype, P_type = *Prevotella* enterotype. ^§^ Model adjusted for maternal birthplace, maternal exposure to psychotropic medications during pregnancy, mother’s pre-pregnancy BMI, gestational age, child delivery mode, mother’s age at birth, child sex, sibling, maternal education at 2 months, household income at 2 months, pet ownership at 2 months, breastfeeding duration, child BMI Z-score at 2 years, main mode of childcare at 2 years, child’s diet at 2 years, exact child’s exact age at stool collection, child tobacco exposure from pregnancy until 3 years, child’s antibiotics intake between 2 and 3 years, and residential setting at 3 years. ^†^ Standardized. ^‡^ *Bacteroides* enterotypes are the references.

**Table 3:** The overall gut microbiota community composition differences and sleep clusters (*n* = 597).

	Crude			Adjusted ^†^		
	R^2^	*p*-Value	FDR *p*-Value	R^2^	*p*-Value	FDR *p*-Value
Beta Diversity distances						
Bray–Curtis	0.0014	0.53	0.53	0.0013	0.62	0.71
Weighted UniFrac	0.0024	0.19	0.19	0.0020	0.34	0.76

FDR = False Discovery Rate. ^†^ Models are adjusted for maternal birthplace, maternal exposure to psychotropic medications during pregnancy, mother’s pre-pregnancy BMI, gestational age, child delivery mode, mother’s age at birth, child sex, siblings, maternal education at 2 months, household income at 2 months, pet ownership at 2 months, breastfeeding, child BMI Z-score at 2 years, main mode of childcare at 2 years, child’s diet at 2 years, child’s exact age at stool collection, child tobacco exposure from pregnancy until 3 years, child’s antibiotics intake between 2 and 3 years, and residential setting at 3 years.

## Data Availability

The data underlying this article cannot be shared publicly due to the privacy of individuals who participated in the study. The data used for this study is available and will be shared at reasonable request to the cohort scientific committee.

## Supplementary Materials

The following supporting information can be downloaded at: https://www.mdpi.com/article/10.3390/children12091240/s1, Table S1: Principle Component Analysis (PCA) factor loadings of the food items reported in the 2-years child dietary questionnaire; Table S2: Differential abundance testing results for genera significantly associated with sleep clusters according to the ANCOMBC analysis; Table S3: Complete-case analysis of the gut microbiota-sleep association (*n* = 374). ‘Optimal’ sleep cluster is the reference; Table S4: The PERMANOVA analysis of variance on the complete-case data (*n* = 374).

## Abbreviations

The following abbreviations are used in this manuscript:

ALDEx2	Analysis of Differential Abundance Taking Sample Variation into Account
ANCOM-BC	Analysis of Compositions of Microbiomes with Bias Correction
BMI	Body Mass Index
DAG	Directed Acyclic Graph
DMM	Dirichlet Multinomial Mixtures
DOHaD	Developmental Origins of Health and Disease
ELFE	Étude Longitudinale Française depuis l’Enfance
FDR	False Discovery Rate
LCA	Latent Class Analysis
NRMSE	Normalized Root Mean Squared Error
OTU	Operational Taxonomic Unit
PAM	Partitioning Around Medoids
PCA	Principal Component Analysis
PCoA	Principal Coordinate Analysis
PERMANOVA	Permutational Multivariate Analysis of Variance
PFC	Proportion of Fractional Change
