# Supplementary material for: Gut Microbiota Composition and Sleep in Preschoolers: The ELFE Birth Cohort Study
Source: Children (Basel). 2025 Sep 16;12(9):1240. doi: 10.3390/children12091240 (PMC12468531; doi:10.3390/children12091240)
Supplement: Supplementary file 1 [file children-12-01240-s001.zip › children-3814447-supplementary.pdf]

Supplementary

Table S1. Principle Component Analysis (PCA) factor loadings of the food items reported in the 2-years child dietary questionnaire

	PCA Factor loadings	
**Food Item**	**Unhealthy dietary pattern**	**Healthy dietary pattern**
**Cereals**	0.02	0.013
**Dairy products**	**0.19**	0.18
**Sugary drinks**	**0.34**	**-0.43**
**Fish**	0.16	0,21
**Chips**	0.22	-0.18
**French fries**	**0.52**	**-0.41**
**Meat and ham**	**0.51**	0.23
**Quiche**	**0.49**	-0.24
**Pastry**	**0.46**	-0.21
**Egg**	**0.40**	0.05
**Charcuterie**	**0.39**	**-0.33**
**Fruit juice**	**0.39**	-0.11
**Cheese**	**0.38**	0.16
**Bread**	**0.37**	0.24
**Sweets**	**0.36**	-0.20
**Fresh fruit**	**0.33**	**0.36**
**Pasta**	0.29	**0.56**
**Raw vegetables**	**0.32**	0.24
**Cooked vegetable**	0.18	**0.74**
**Fruit compote**	0.05	**0.30**

Factor loadings correspond to the correlation of food items with a given dietary pattern. Factor loadings colored in bold (> 0.30 or < 0.30) correspond to the threshold we defined for a positive > 0.30 or negative < 0.30 contribution to the dietary pattern

Table S2. Differential abundance testing results for genera significantly associated with sleep clusters according to the ANCOMBC analysis.

raw.pvalue	adj.pvalue	lfc	otu.id
**0.0001468598**‡	0.01615457	-0.3361305	Bacteria∣Firmicutes∣Clostridia∣Oscillospirales∣Ruminococcaceae∣unknown genus
**0.0007873563**	0.04330460	-0.2842330	Bacteria∣Firmicutes∣Bacilli∣Erysipelotrichales∣Erysipelatoclostridiaceae∣Erysipelatoclostridium

adj.pvalue: False discovery rate (FDR) adjusted p-value, lfc: log fold change, ‡Reference.

Model adjusted for maternal birthplace, maternal exposure to psychotropic medications during pregnancy, mother’s pre-pregnancy BMI, gestational age, child delivery mode, mother’s age at birth, child sex, sibling, maternal education at 2 months, household income at 2 months, pet ownership at 2 months, breastfeeding duration, child BMI Z-score at 2 years, main mode of childcare at 2 years, child’s diet at 2 years, child age at stool collection, child tobacco exposure from pregnancy until 3 years, child’s antibiotics intake between 2 and 3 years, residential setting at 3 years

Table S3. Complete-case analysis of the gut microbiota-sleep association (n = 374). 'Optimal' sleep cluster is the reference

	Crude			Adjusted [š]		
	**OR**	**95% CI**	**P-value**	**OR**	**95% CI**	**P-value**
**Alpha diversity metrics**						
**Chao1 estimate[†]**	1.05	0.83 - 1.33	0.67	1.01	0.78 -1.32	0.93
**Shannon index[†]**	1.13	0.90 - 1.44	0.30	1.04	0.80 -1.37	0.76
**Enterotypes**						
**B_type[‡]**	—	—	0.45	—	—	0.58
**P_type**	1.28	0.66 - 2.39		1.22	0.59 - 2.43	

OR = Odds Ratio, CI = Confidence Interval, B_type = *Bacteroides* enterotype, P_type = *Prevotella* enterotype

[š] Model adjusted for maternal birthplace, maternal exposure to psychotropic medications during pregnancy, mother's pre-pregnancy BMI, gestational age, child delivery mode, mother's age at birth, child sex, sibling, maternal education at 2 months, household income at 2 months, pet ownership at 2 months, breastfeeding duration, child BMI Z-score at 2 years, main mode of childcare at 2 years, child's diet at 2 years, child age at stool collection, child tobacco exposure from pregnancy until 3 years, child's antibiotics intake between 2 and 3 years, residential setting at 3 years

[†]Standardized

[‡]*Bacteroides* enterotypes is the reference

Table S4. The PERMANOVA analysis of variance on the complete-case data (n = 374)

	**Crude**			**Adjusted [š]**		
	**R^2**	**P-value**	**FDR P-value**	**R^2**	**P-value**	**FDR P-value**
**Beta Diversity metrics**						
Bray-Curtis	0.002	0.50	0.50	0.003	0.45	0.75
Weighted UniFrac	0.004	0.16	0.16	0.003	0.22	0.62

FDR = False Discovery Rate

š Model adjusted for maternal birthplace, maternal exposure to psychotropic medications during pregnancy, mother's pre-pregnancy BMI, gestational age, child delivery mode, mother's age at birth, child sex, sibling, maternal education at 2 months, household income at 2 months, pet ownership at 2 months, breastfeeding duration, child BMI Z-score at 2 years, main mode of childcare at 2 years, child's diet at 2 years, exact child's age at stool collection, child tobacco exposure from pregnancy until 3 years, child's antibiotics intake between 2 and 3 years, residential setting at 3 years
